# Influence of Chloroplast Defects on Formation of Jasmonic Acid and Characteristic Aroma Compounds in Tea (*Camellia sinensis*) Leaves Exposed to Postharvest Stresses

**DOI:** 10.3390/ijms20051044

**Published:** 2019-02-27

**Authors:** Jianlong Li, Lanting Zeng, Yinyin Liao, Dachuan Gu, Jinchi Tang, Ziyin Yang

**Affiliations:** 1 Tea Research Institute, Guangdong Academy of Agricultural Sciences & Guangdong Provincial Key Laboratory of Tea Plant Resources Innovation and Utilization, Dafeng Road 6, Tianhe District, Guangzhou 510640, China; skylong.41@163.com; 2 Guangdong Provincial Key Laboratory of Applied Botany & Key Laboratory of South China Agricultural Plant Molecular Analysis and Genetic Improvement, South China Botanical Garden, Chinese Academy of Sciences, Xingke Road 723, Tianhe District, Guangzhou 510650, China; zenglanting@scbg.ac.cn (L.Z.); honey_yyliao@scbg.ac.cn (Y.L.); gdcawang@126.com (D.G.); 3 College of Advanced Agricultural Sciences, University of Chinese Academy of Sciences, No. 19A Yuquan Road, Beijing 100049, China

**Keywords:** albino, aroma, *Camellia sinensis*, chloroplast, jasmonic acid, light-sensitive, stress, tea, volatile

## Abstract

Characteristic aroma formation in tea (*Camellia sinensis*) leaves during the oolong tea manufacturing process might result from the defense responses of tea leaves against these various stresses, which involves upregulation of the upstream signal phytohormones related to leaf chloroplasts, such as jasmonic acid (JA). Whether chloroplast changes affect the formation of JA and characteristic aroma compounds in tea leaves exposed to stresses is unknown. In tea germplasms, albino-induced yellow tea leaves have defects in chloroplast ultrastructure and composition. Herein, we have compared the differential responses of phytohormone and characteristic aroma compound formation in normal green and albino-induced yellow tea leaves exposed to continuous wounding stress, which is the main stress in oolong tea manufacture. In contrast to single wounding stress (from picking, as a control), continuous wounding stress can upregulate the expression of *CsMYC2*, a key transcription factor of JA signaling, and activate the synthesis of JA and characteristic aroma compounds in both normal tea leaves (normal chloroplasts) and albino tea leaves (chloroplast defects). Chloroplast defects had no significant effect on the expression levels of *CsMYC2* and JA synthesis-related genes in response to continuous wounding stress, but reduced the increase in JA content in response to continuous wounding stress. Furthermore, chloroplast defects reduced the increase in volatile fatty acid derivatives, including jasmine lactone and green leaf volatile contents, in response to continuous wounding stress. Overall, the formation of metabolites derived from fatty acids, such as JA, jasmine lactone, and green leaf volatiles in tea leaves, in response to continuous wounding stress, was affected by chloroplast defects. This information will improve understanding of the relationship of the stress responses of JA and aroma compound formation with chloroplast changes in tea.

## 1. Introduction

Plants generally synthesize large amounts of volatile compounds in response to environmental stresses. These volatile metabolites contribute to plant defense against environmental stresses [1,2] and can also be regarded as important quality components in crops [3]. Utilizing the stress response to improve the natural quality components of horticultural crops has recently attracted increasing attention. Tea (*Camellia sinensis*) plants are famous horticultural and representative plants in China, with their leaves used to make the second most popular beverage, globally, after water. Aroma is an important factor affecting the character and quality of tea [4]. Aroma formation in tea leaves can result from the defense responses of tea leaves against various stresses during the preharvest (tea growth) and postharvest (tea manufacture) processes [3,4]. During tea growth, attack by insects, such as tea green leafhoppers [5,6], and light conditions, such as dark, blue-light, and red-light treatments [7,8], can significantly increase endogenous aroma compounds or produce new aroma compounds in tea leaves. During the tea manufacturing process, especially that of oolong tea, tea leaves are exposed to various stresses, including plucking (wounding), solar withering (drought, heat, and UV radiation), indoor withering (drought), and turnover (continuous wounding) [5,9]. Aroma formation in tea leaves during the oolong tea manufacturing process might result from the defense responses of tea leaves against these various stresses [3]. For example, levels of three characteristic aroma compounds, including indole, jasmine lactone, and (*E*)-nerolidol, were significantly enhanced during the turnover stage of oolong tea manufacture owing to continuous wounding stress [9,10,11,12,13]. As these aroma compounds are biosynthesized through different metabolic pathways, it has been proposed that common upstream signals regulate the multiple biosynthetic pathways of tea aromas under these stresses [3]. Phytohormones, especially jasmonic acid (JA), have been reported to regulate the biosynthesis of volatiles and act as important upstream signal chemicals [14]. Several studies have shown that JA is involved in the formation of tea aroma compounds [15,16]. In some plants, the main upstream synthetic pathways involved in JA formation (linoleic acid (LA) is released from chloroplast lipids, then converted to 12-oxo-phytodienoic acid by lipoxygenases (LOX), allene oxide synthase (AOS), and allene oxide cyclase (AOC) as catalysts) have been reported to mostly occur in the chloroplast [17,18,19,20]. In our previous study, CsAOS2 isolated from *Camellia sinensis* was found to have a role in JA synthesis, and was located in the chloroplast membrane [21]. This suggested that chloroplasts might be related to JA synthesis in tea. This inspired our interest in whether chloroplast defects affect JA synthesis and, consequently, aroma formation in tea leaves. 

Generally, tea cultivars naturally grown as green leaves are usually processed into tea products. However, tea mutants whose young shoots are grown in white or yellow color in some conditions have recently attracted a significant increasing attention from the researchers and manufactures in the related areas, because they are supposed as potential raw materials to be processed into “high-quality” tea products [22]. Light-sensitive and low-temperature-sensitive types are mainly albino teas, and their new grown shoots exhibit white or yellow color [23,24]. In light-sensitive mutants, strong light illumination is a determinant factor, while in low-temperature-sensitive types, low temperature is a prerequisite [25,26,27]. Former studies showed that a significant deficiency of chlorophylls mainly result in white or yellow shoots in these albino tea mutants [23,24,28]. In some recent studies, the samples used to investigate the underlying mechanism of albino tea leaves are under different genetic background, geography, climate, amongst other factors. Therefore, to avoid the interference from these factors, *C. sinensis* cv. Yinghong No. 9 (an original cultivar grown with green leaves), and its light-sensitive mutant (albino-induced yellow leaves in strong light illumination) were collected in pairs under the same growth conditions as used in our previous experiments [22,28]. This light-sensitive mutant shared the same genetic background of the original cultivar. Under no-stress conditions, the freshly picked albino-induced yellow tea leaves had lower contents of free tea aroma compounds than the freshly picked normal green tea leaves [22]. Furthermore, the albino-induced yellow tea leaves had defects in the chloroplast ultrastructure and composition [28]. This allowed us to use albino-induced yellow tea leaves as a model for studying the influence of chloroplast defects on the formation of characteristic aroma compounds in tea leaves exposed to continuous wounding stress, which is a major stress in the oolong tea manufacturing process. In this study, we compared the endogenous free tea aroma compound contents of yellow leaves and green leaves either under single wounding stress (from picking, as a control) or continuous wounding stress (from tea manufacture). Furthermore, expression levels of key synthetic genes involved in the formation of characteristic aroma compounds in both tea leaves under continuous wounding stress were investigated. Finally, the tea aroma formation-related upstream signals in both tea leaves under continuous wounding stress were studied. 

## 2. Results 

### 2.1. Effect of Chloroplast Defects on Characteristic Aroma Compound Contents in Response to Continuous Wounding Stress

**Figure 1:**
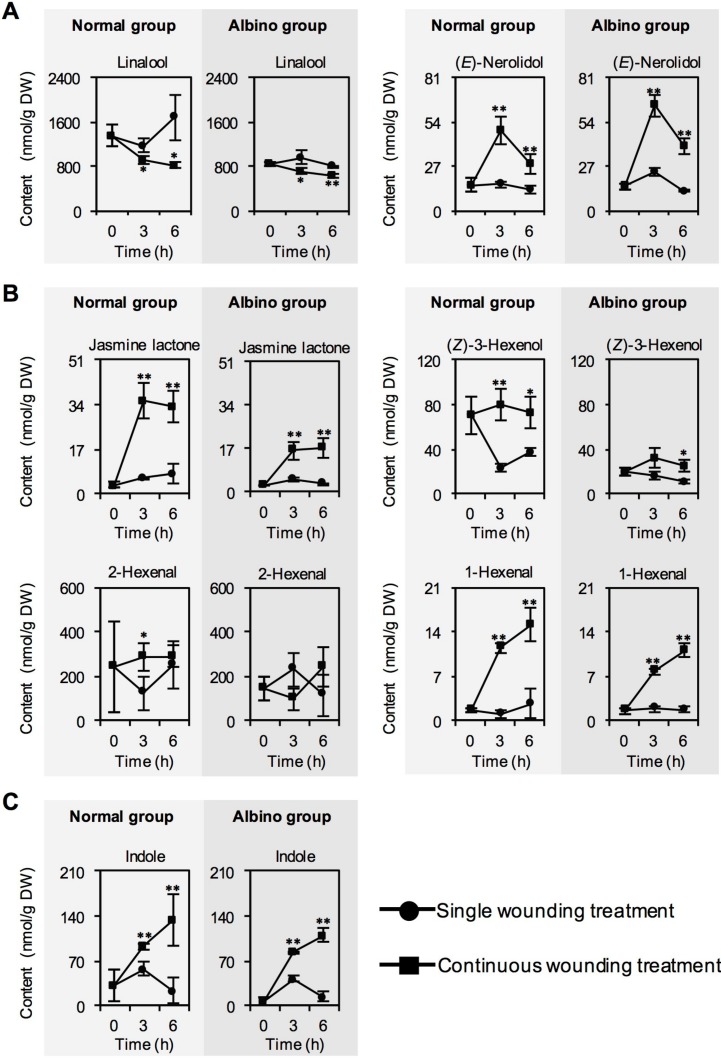
Changes in contents of characteristic aroma compound biosynthetic genes of normal tea leaves and albino tea leaves exposed to single wounding treatment and continuous wounding treatment, respectively. Data are expressed as mean ± SD (*n* = 3). * *p* ≤ 0.05; **, *p* ≤ 0.01, comparison between single wounding treatment and continuous wounding treatment at the same treatment time. (**A**) Volatile terpenes. (**B**) Volatile fatty acid derivatives. (**C**) Indole.

We first investigated whether chloroplast defects affected the characteristic aroma compound contents in response to continuous wounding stress. The mass chromatogram of identified aroma compounds is shown in Figure S1. When tea leaves are picked, they are exposed to a single wounding stress as one main stress. Therefore, picked tea leaves left to stand for a certain time were the single wounding stress treatment group used as a control. Stress-induced aroma compound formation mainly occurs during the turnover stage of oolong tea manufacturing process, which involves continuous wounding stress. A shaking machine was employed to simulate continuous wounding stress from the turnover stage. Tea leaves subjected to this processing were regarded as the continuous wounding stress treatment group. In contrast to single wounding stress, continuous wounding stress upregulated the contents of (*E*)-nerolidol, jasmine lactone, and green leaf volatiles (including (*Z*)-3-hexenol, 1-hexenal, and 2-hexenal), and indole, and reduced the linalool content (Figure 1). Chloroplast defects reduced the accumulation of volatile fatty acid derivatives, including jasmine lactone and green leaf volatiles (Figure 1B), but had no significant effect on (*E*)-nerolidol and indole accumulation in response to continuous wounding stress (Figure 1A,C).

### 2.2. Effect of Chloroplast Defects on Expression Levels of Characteristic Genes for Aroma Compound Biosynthesis in Response to Continuous Wounding Stress

**Figure 2:**
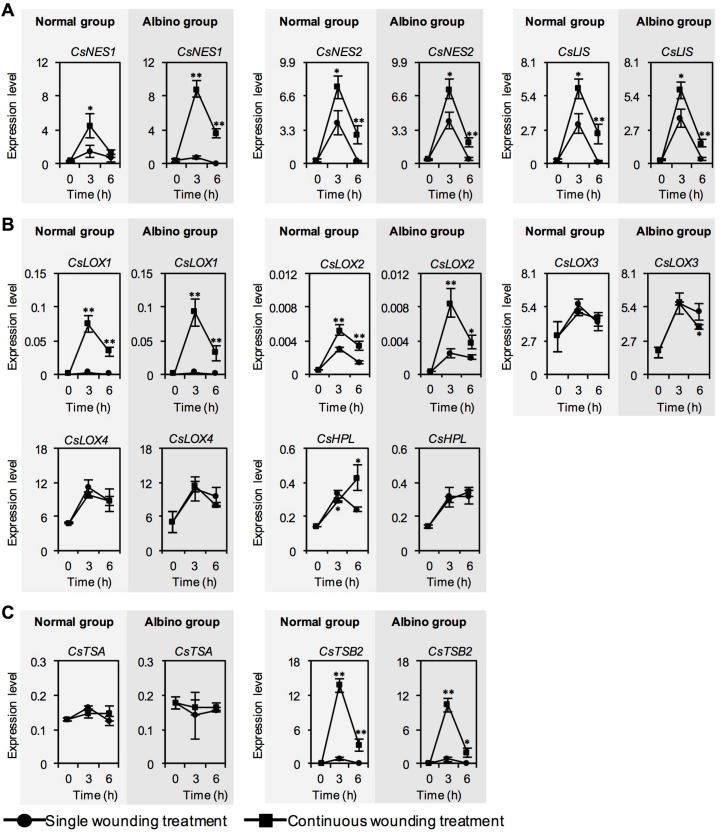
Changes in expression level of characteristic aroma compound biosynthetic genes of normal tea leaves and albino tea leaves exposed to single wounding treatment and continuous wounding treatment, respectively. Data are expressed as mean ± SD (*n* =3). * *p* ≤ 0.05; ** *p* ≤ 0.01, comparison between single wounding treatment and continuous wounding treatment at the same treatment time. *NES*, *(E)-nerolidol synthase*; *LIS*, *linalool synthase*; *LOX*, *lipoxygenase*; *HPL*, *hydroperoxide lyase*; *TSA*, *tryptophan synthase α-subunit*; *TSB*, *tryptophan synthase β-subunit*. (**A**) Genes involved in formations of volatile terpenes. (**B**) Genes involved in formations of volatile fatty acid derivatives. (**C**) Genes involved in formation of indole.

We next investigated whether chloroplast defects affected the expression levels of characteristic genes for aroma compound biosynthesis in response to continuous wounding stress. In contrast to single wounding stress, continuous wounding stress upregulated the expression levels of key characteristic genes for aroma compound biosynthesis, including *CsNES1* and *CsNES2* (responsible for (*E*)-nerolidol synthesis), *CsLIS* (responsible for linalool synthesis), *CsLOX1*, *CsLOX2*, and *CsHPL* (responsible for jasmine lactone and green leaf volatile syntheses), and *CsTSB2* (responsible for indole synthesis). No significant difference was observed in the continuous wounding response patterns of most characteristic genes for aroma compound biosynthesis between normal tea leaves and albino tea leaves, while *CsHPL* expression was upregulated only in normal tea leaves exposed to continuous wounding stress (Figure 2B). This suggested that chloroplast defects affected *CsHPL* expression, which might lead to less accumulation of green leaf volatiles in albino tea leaves exposed to continuous wounding stress (Figure 1).

### 2.3. Effect of Chloroplast Defects on Phytohormone Contents in Response to Continuous Wounding Stress

**Figure 3:**
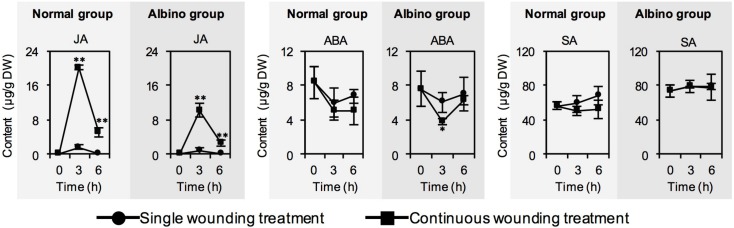
Changes in contents of phytohormones of normal tea leaves and albino tea leaves exposed to single wounding treatment and continuous wounding treatment, respectively. Data are expressed as mean ± SD (*n* =3). * *p* ≤ 0.05; ** *p* ≤ 0.01, comparison between single wounding treatment and continuous wounding treatment at the same treatment time. JA, jasmonic acid; ABA, abscisic acid; SA, salicylic acid.

We also investigated whether chloroplast defects affected phytohormone contents in response to continuous wounding stress. In contrast to single wounding stress, continuous wounding stress significantly increased the JA content, but had no significant effect on abscisic acid (ABA) and salicylic acid (SA) contents both in normal tea leaves and albino tea leaves (Figure 3). Furthermore, chloroplast defects reduced the increase in JA content in response to continuous wounding stress, although there was no significant difference between the continuous wounding response patterns of phytohormone contents in normal tea leaves and albino tea leaves (Figure 3).

### 2.4. Effect of Chloroplast Defects on Expression Levels of JA Synthesis-Related Genes in Response to Continuous Wounding Stress

**Figure 4:**
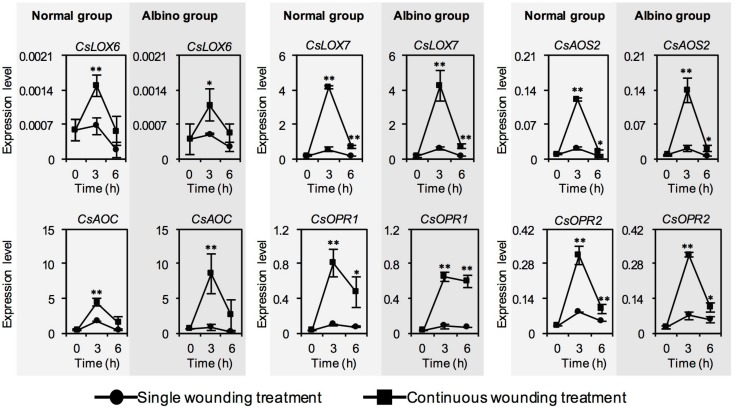
Changes in expression level of jasmonic acid (JA) biosynthetic genes of normal tea leaves and albino tea leaves exposed to single wounding treatment and continuous wounding treatment, respectively. Data are expressed as mean ± SD (*n* =3). * *p* ≤ 0.05; ** *p* ≤ 0.01, comparison between single wounding treatment and continuous wounding treatment at the same treatment time. *LOX*, *lipoxygenase*; *AOS*, *allene oxide synthase*; *AOC*, *allene oxide cyclase*; *OPR*, *12-oxo-phytodienoic acid reductase*.

As the JA content was significantly increased by continuous wounding stress both in normal tea leaves and albino tea leaves, we investigated whether chloroplast defects affected the expression levels of JA synthesis-related genes in response to continuous wounding stress. In contrast to single wounding stress, continuous wounding stress upregulated expression levels of most genes involved in JA synthesis, both in normal tea leaves and albino tea leaves, with no significant difference between the continuous wounding response patterns in normal tea leaves and albino tea leaves (Figure 4). This suggested that chloroplast defects did not significantly affect expression levels of JA synthesis-related genes in response to continuous wounding stress.

### 2.5. Effect of Chloroplast Defects on Expression Level of CsMYC2, a Key Transcription Factor of JA Signaling, in Response to Continuous Wounding Stress

**Figure 5:**
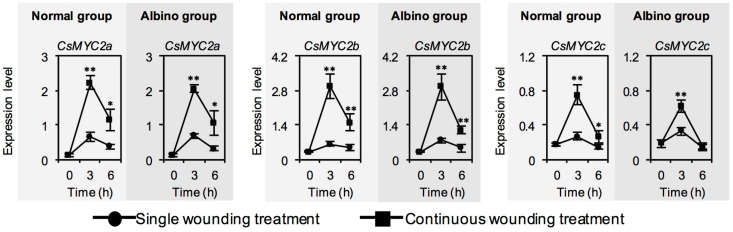
Changes in expression level of *CsMYC2*, a key transcription factor of jasmonic acid (JA), of normal tea leaves and albino tea leaves exposed to single wounding treatment and continuous wounding treatment, respectively. Data are expressed as mean ± SD (*n* =3). * *p* ≤ 0.05; ** *p* ≤ 0.01, comparison between single wounding treatment and continuous wounding treatment at the same treatment time.

We next investigated whether chloroplast defects affected the expression levels of *CsMYC2*, a key transcription factor of JA signaling, in response to continuous wounding stress. In contrast to single wounding stress, continuous wounding stress upregulated expression levels of *CsMYC2*, both in normal tea leaves and albino tea leaves, and no significant difference was observed between the continuous wounding response patterns in normal tea leaves and albino tea leaves (Figure 5). This suggested that chloroplast defects did not significantly affect the expression levels of *CsMYC2* in response to continuous wounding stress.

## 3. Discussion

Recently, using stress responses to improve tea aromas has attracted increased attention [3]. This approach is not only related to the tea growth process, but also explains aroma formation during the tea manufacturing process. Among the six types of tea, aroma formation during the oolong tea manufacturing process is a representative example of stress-induced tea aroma, because oolong tea manufacture involves the most stresses. Among stresses applied in the oolong tea manufacturing process, continuous wounding in the turnover stage significantly affects the high accumulation of tea aroma compounds, which contributes to the floral odor of oolong tea [3,9,10,11,13]. Continuous wounding can induce the high accumulation of characteristic aroma compounds, such as indole, jasmine lactone, and (*E*)-nerolidol. Furthermore, these compounds are produced by the activation of key aroma synthetic genes in response to continuous wounding stress [10,11,13]. These observations were made using normal green tea leaves with normal chloroplasts. In this study, normal tea leaves with normal chloroplasts showed similar tea aroma formation results in response to continuous wounding stress (Figure 1), although a different cultivar (cv. Yinghong No. 9) was used. This suggested that the relationship between continuous wounding stress and tea aroma formation occurs widely in different tea cultivars. In the present study, chloroplast was partly defective in the light-sensitive mutant (albino-induced yellow) of cv. Yinghong No. 9 original cultivar (green) [28]. Microscopy observations indicated that the albino tea leaves exhibited a significant reduction in number of chloroplasts, and some chloroplasts showed grana thylakoid structures that were damaged or developing (called etioplasts) [28]. This mutant was used to investigate whether chloroplast defects affected tea aroma compound formation in response to continuous wounding stress. The results showed that chloroplast defects did not significantly affect the formation of indole and (*E*)-nerolidol (Figure 1 and Figure 2). Furthermore, the linalool content was not increased by continuous wounding stress both in normal tea leaves and albino tea leaves (Figure 1A), although expression of its synthetic gene, *CsLIS*, was significantly upregulated by continuous wounding stress both in normal tea leaves and albino tea leaves (Figure 2A). The change in linalool formation under continuous wounding stress showed a similar trend to that during the turnover stage of the oolong tea manufacturing process [9,12]. However, it is yet to be determined whether these changes are related to linalool glycosidation, metabolism, or emission. Notably, chloroplast defects reduced the accumulation of volatile fatty acid derivatives, including jasmine lactone and green leaf volatiles, in response to continuous wounding stress (Figure 1B). In general, the final synthetic steps of these volatiles occur in the cytosol [29], but their formations were affected by chloroplast defects in tea leaves. Therefore, it would be interesting to investigate whether CsHPL located in the cytosol is affected by chloroplast defects and, consequently, affects green leaf volatile formation.

In the investigations of *C. sinensis*, a mature genetic transformation system has not been firmly established. Therefore, most genes related to the formation of tea aroma compounds have not been functionally characterized, in vivo, in tea plants over the past decade [3]. These genes were usually expressed in *Escherichia coli*, yeast, or an insect cell system. In addition, some of these genes were further functionally characterized in transient overexpression model plant systems [3]. Many genes involved in the final biosynthetic step of several important aroma compounds, including (*S*)-linalool, (*E*)-nerolidol, and indole, have been functionally characterized, and the related contents have been summarized in our previous review [3]. *CsTPSs* are responsible for the formation of (*S*)-linalool and (*E*)-nerolidol, and *CsTSA* and *CsTSBs* play roles in indole synthesis in tea. *CsNES1* and *CsNES2*, identified in the present study (Figure 2A), have been proposed to be responsible for (*E*)-nerolidol formation based on their subcellular localization in the cytosol and function validation in *Escherichia coli* and transient overexpression model plant systems [11,30]. *CsLIS*, also found in the present study (Figure 2A), was proposed to be responsible for (*S*)-linalool formation based on its subcellular localization in plastids and functional validation in *Escherichia coli* and transient overexpression model plant systems [7,30]. Our previous study showed that the protein mixture of CsTSA (tryptophan synthase α-subunit) and CsTSB2 (tryptophan synthase β-subunit) catalyzes indole formation in vitro [10], suggesting that CsTSA and CsTSB2 might be a protein complex in *C. sinensis* that is similar to the α_2_β_2_ tetramer in bacteria [31]. In the present study, only *CsTSB2* was significantly upregulated by continuous wounding stress both in normal tea leaves and albino tea leaves, while *CsTSA* was not significantly affected (Figure 2C), suggesting that *CsTSB2* was more sensitive to wounding stress compared with *CsTSA*. Furthermore, a few enzymes and genes involved in the upstream pathways responsible for tea aroma compound formation, such as CsLOX1 for jasmine lactone formation [13], have also been functionally characterized. Based on correlation analysis of *CsLOXs* gene expression and the stresses and tissue distributions of expression of different *CsLOX* genes, *CsLOX1*, *CsLOX2*, *CsLOX3*, and *CsLOX4*, found in the present study (Figure 2B), were proposed to be closely involved in the syntheses of green leaf volatiles [32]. *CsHPL*, found in the present study (Figure 2B), has also been proposed to be involved in green leaf volatile synthesis based on function validation in *E. coli* [33], because functional identification of *CsHPL* showed that recombinant CsHPL can catalyze 13-hydroperoxy-9(*Z*), 11(*E*), 15(*Z*)-octadecatrienoic acid into 3-(*Z*)-hexenal, which is a key precursor of 3-(*Z*)-hexenol, 2-(*Z*)-hexenal, and 2-(*Z*)-hexenol [34]. In the present study, the levels of key genes involved in tea aroma formation were investigated to compare the tea aroma biosynthesis abilities of albino-induced yellow leaves and normal green tea leaves exposed to wounding stress (Figure 2). As the functions of most genes involved in tea aroma formation investigated in this study have been validated previously, the differences in metabolic flux between albino-induced yellow leaves and normal green tea leaves were more reliable.

Phytohormones are key upstream signals, especially in regulating the formation of plant volatiles in response to environmental stresses. Among phytohormones, JA has mostly been reported to be related to plant volatiles. In contrast to phytohormone formation in preharvest tea leaves exposed to stresses, little is known about phytohormone formation under postharvest stresses. This study found that continuous wounding stress from postharvest tea processing had no significant effect on the ABA and SA contents, but increased the JA content both in normal tea leaves and albino tea leaves (Figure 3), which was attributed to activation of the expression of most JA synthetic genes (Figure 4). Furthermore, chloroplast defects reduced the increase in JA content in response to continuous wounding stress (Figure 3). Current knowledge concerning phytohormone and metabolite biosynthesis in tea plants is mostly based on findings reported for other plant species. Based on correlation analysis of expression of different *CsLOX* genes and stresses, and tissue distributions of expression of different *CsLOX* genes, *CsLOX1* (Figure 2), *CsLOX6*, and *CsLOX7* (Figure 4) were proposed to be closely involved in JA synthesis [32]. Furthermore, using a transient expression system in *Nicotiana benthamiana* plants, *CsAOS2*, a gene involved in JA synthesis, was validated to have a function in JA synthesis and located in the chloroplast membrane [21]. MYC2 is the core transcription factor of JA signaling. Three *Arabidopsis MYC2* homologue genes were found in the *C. sinensis* genome database (Figure 5). Among these three MYC2s, CsMYC2a was clustered together with the reported functional MYC2 in *Arabidopsis* [35]. In the study, the expression levels of *CsMYC2* were not significantly affected by chloroplast defection in the albino leaves. Besides *CsMYC2* and JA synthetic genes, other factors—such as precursor metabolites—involved in JA synthesis may affect JA synthesis in the albino leaves under wounding stress, since other metabolites that are shared with the partly fatty acid-derived pathways of JA, such as jasmine lactone and green leaf volatiles, had similar effects resulting from chloroplast defects (Figure 1B). As authentic standards of many precursors of JA synthesis are unavailable, we did not confirm this hypothesis in the present study. In future work, we will try to obtain authentic standards of the key precursors of JA synthesis and confirm this hypothesis. In the present study, although the precursor of ABA is also synthesized in plastid, ABA biosynthesis was not significantly influenced by chloroplast defects (Figure 3). Wounding stress was the main stress in the treatments of the present study. Compared with ABA synthesis, JA synthesis was generally more sensitive to the wounding stress. It remains to be determined whether ABA biosynthesis is influenced by chloroplast defection under other stresses such as drought stress.

## 4. Materials and Methods 

### 4.1. Plant Materials and Treatments

One bud and three leaves of *C. sinensis* cv. Yinghong No. 9 and its yellow mutant (a light-sensitive variant) were plucked and used in the present study. These tea samples were picked at the Tea Research Institute, Guangdong Academy of Agricultural Sciences (Yingde, Guangdong, China), in November 2018. 

The plucked tea leaves were shaken using a shaking table at 23 ± 1 °C and 60% humidity, and collected after continuous shaking for 0, 3, and 6 h (continuous wounding treatment). The tea leaves, without continuous shaking, stored under the same conditions for 0, 3, and 6 h were used as controls (single wounding treatment). After treatment, the samples were frozen immediately with liquid nitrogen and stored at –80 °C for further study.

### 4.2. Extraction and Analysis of Aroma Compounds in Tea Leaves

According to our previous studies [12,13], direct organic solvent extraction was applied to investigate content changes in endogenous aroma compounds of finely powered tea leaves [12,13]. Dichloromethane (1.8 mL) was used to extract aroma compounds from 300 mg (fresh weight) of tea samples, and 5 nmol of ethyl decanoate was added to the organic solvent as an internal standard. The mixture was treated in a shaker at room temperature, and the extract was collected after overnight extraction. Anhydrous sodium sulfate was used to dry the extract, and nitrogen was applied to condense the extract into a 200 μL volume. The extract (1 μL) was then subjected to gas chromatography–mass spectrometry (GC–MS) analysis conforming on a GC–MS QP2010 SE (Shimadzu Corporation, Kyoto, Japan) equipped with GCMS Solution software (Version 2.72, Shimadzu Corporation, Japan). The sample was injected in splitless mode for 1 min under 230 °C of GC port. A SUPELCOWAX 10 column (30 m × 0.25 mm × 0.25 μm, Supelco Inc., Bellefonte, PA, USA) was used to separate the aroma compounds, with helium as a carrier gas in the flow rate of 1 mL/min. Initially, the temperature of GC oven was kept at 60 °C for 3 min, then ramped to 240 °C at 4 °C/min, and held for another 20 min at 240 °C. A full scan mode was applied, and the mass spectrometry ranged from *m/z* 40 to *m/z* 200. The authentic standards were used to make the identification of aroma compounds, and quantitative analyses of compounds were constructed according to the calibration curves by plotting the concentration against the peak area of the authentic standard [22]. The dry weight of the sample was calculated based on the weight of fresh leaves and dried leaves obtained after drying.

### 4.3. Transcript Expression Analysis of the Related Genes

Quick RNA Isolation Kit (Huayueyang Biotechnology Co., Ltd., Beijing, China) was used to isolate total RNA from tea samples. The obtained RNA was purified after the removal of genomic DNA (gDNA) and reversely transcribed into cDNA using PrimeScript RT Reagent Kit with gDNA Eraser (Takara Bio Inc., Kyoto, Japan). Quantitative real time PCR (qRT-PCR) were applied to analyze the transcript expression level of gene [10,12,13]. The reaction system (20 μL) contained 10 μL iTaq^TM^ Universal SYBR^®^ Green Supermix (Bio-Rad, Hercules, CA, USA), 0.4 μL of each specific primer (10 μM), 2 μL cDNA (diluted into 20-fold), and 7.2 μL ddH_2_O. The qRT-PCR analysis was performed on a Roche LightCycle 480 (Roche Applied Science, Mannheim, Germany). One cycle of 95 °C for 60 s, and 40 cycles of 95 °C for 15 s and 60 °C for 30 s were used as PCR conditions. At the end of each PCR reaction, to verify the specificity of PCR product, a melt curve was carried out. Calculation of the relative expression level of genes was according to the 2^−^^∆∆*C*t^ method, and based on the normalization to mRNA level of the reference gene. Identification and evaluation of reliable reference genes for qRT-PCR analysis in tea plants showed that encoding elongation factor1 (*CsEF1*) was the most stable reference gene in diurnal expression series [36]. In the study, the samples were collected during 6 h, and *CsEF1* was used as a reference gene. The primers used for qRT–PCR analysis are provided in Table S1.

### 4.4. Analysis of Phytohormone Contents in Tea Leaves

Finely powdered sample (300 mg, fresh weight) was extracted with 3 mL ethyl acetate by vortexing for 30 s followed by ultrasonic extraction in ice-cold water for 20 min. [^2^H_5_]JA, [^2^H_4_]SA, and [^2^H_6_]ABA were added to the mixture as internal standards. After centrifuging at 10,000× *g* for 5 min at 4 °C, 2.9 mL supernatants were collected and then dried under a stream of nitrogen. The residue was re-dissolved in 200 μL methanol. The supernatants were filtered through a 0.22 μm membrane, and subjected to an ultra performance liquid chromatography/quadrupole time-of-flight mass spectrometry (UPLC–QTOF-MS) (Acquity UPLC I-Class/ Xevo® G2-XS QTOF, Waters Corporation, MA, USA). Each sample (5 μL) was injected onto a Waters ACQUITY UPLC HSS T3 C18 column (2.1 mm × 100 mm, 1.8 μm). Solvent A was Milli-Q water with 0.1% (*v*/*v*) formic acid. Solvent B was acetonitrile with 0.1% (v/v) formic acid. The solvent gradient was started at 20% B, then linearly increased to 35% within 10 min, and later increased to 95% B in 0.1 min and kept for 3 min. In that moment, it suddenly dropped to 20% in 0.1 min and was maintained for 3 min. The flow rate was 0.4 mL/min. The column temperature was 30 °C. The electrospray ionization operated on negative mode. The MS conditions were capillary voltage: 1.5 kV; source temperature: 100 °C; desolvation temperature: 300 °C; cone gas flow: 50 L/h; and desolvation gas flow: 600 L/h. The quantitative analyses of phytohormones were based on calibration curves, which were constructed by plotting the concentration of each phytohormone against the peak area of the authentic standard.

### 4.5. Statistical Analysis

Statistical analysis was performed using SPSS software, version 18.0 (SPSS Inc., Chicago, IL, USA). Two-tailed student’s *t* test was used to determine the differences between the single wounding and continuous wounding treatment. 

## 5. Conclusions

**Figure 6:**
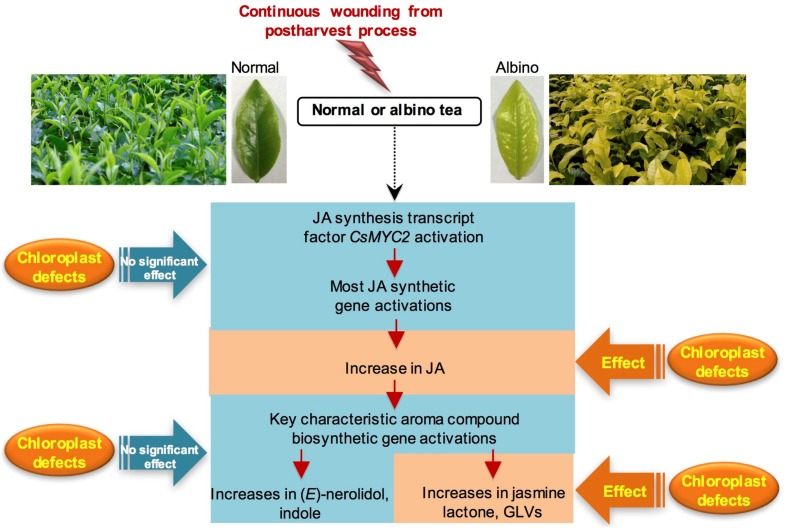
Formations of jasmonic acid (JA) and characteristic aroma compounds in normal tea leaves and albino tea leaves exposed to continuous wounding stress. GLVs, green leaf volatiles.

In the present study, continuous wounding stress, which is a major stress in the postharvest tea manufacturing process, can upregulate expression of *CsMYC2*, a key transcription factor of JA signaling, and activate the synthesis of JA and characteristic aroma compounds (including (*E*)-nerolidol, indole, jasmine lactone, and green leaf volatiles) either in normal tea leaves (normal chloroplasts) or albino tea leaves (chloroplast defects). Furthermore, chloroplast defects did not significantly affect the expression levels of JA synthesis-related genes and *CsMYC2* in response to continuous wounding stress, but reduced the increase in JA content in response to continuous wounding stress. Furthermore, chloroplast defects reduced the increase in volatile fatty acid derivatives, including jasmine lactone and green leaf volatile contents, in response to continuous wounding stress. Overall, the formation of metabolites derived from fatty acids, such as JA, jasmine lactone, and green leaf volatiles in tea leaves, in response to continuous wounding stress, were affected by chloroplast defects (Figure 6). Although fresh albino tea leaves contained relatively low contents of aroma compounds compared with fresh normal tea leaves [22], the stress responses of aroma compounds occurred regardless of chloroplast defects. The information presented here will improve understanding of the relationship between stress responses of phytohormones and aroma compounds, and chloroplast changes. Furthermore, these results provide essential information for the future utilization of stress responses to improve the weak aroma quality of albino tea leaves.

## Supplementary Materials

Supplementary materials can be found at https://www.mdpi.com/1422-0067/20/5/1044/s1.

## Abbreviations

ABA	abscisic acid
AOC	allene oxide cyclase
AOS	allene oxide synthase
EF1	encoding elongation factor 1
GC–MS	gas chromatography–mass spectrometry
HPL	hydroperoxide lyase
JA	jasmonic acid
LIS	linalool synthase
LOX	lipoxygenase
NES	(E)-nerolidol synthase
OPR	12-oxo-phytodienoic acid reductase
SA	salicylic acid
TSA	tryptophan synthase α-subunit
TSB	tryptophan synthase β-subunit
UPLC–QTOF-MS	ultra performance liquid chromatography/quadrupole time-of-flight mass spectrometry

## References

[1] Pichersky E., Gershenzon J. (2002). The formation and function of plant volatiles: Perfumes for pollinator attraction and defense. Curr. Opin. Plant Biol..

[2] Dong F., Fu X.M., Watanabe N., Su X.G., Yang Z.Y. (2016). Recent advances in the emission and functions of plant vegetative volatiles. Molecules.

[3] Zeng L.T., Watanabe N., Yang Z.Y. (2018). Understanding the biosyntheses and stress response mechanisms of aroma compounds in tea (*Camellia sinensis*) to safely and effectively improve tea aroma. Crit. Rev. Food Sci..

[4] Yang Z.Y., Baldermann S., Watanabe N. (2013). Recent studies of the aroma compounds in tea. Food Res. Int..

[5] Cho J.Y., Mizutani M., Shimizu B., Kinoshita T., Ogura M., Tokoro K., Lin M.L., Sakata K. (2007). Chemical profiling and gene expression profiling during the manufacturing process of Taiwan oolong tea “Oriental Beauty”. Biosci. Biotechnol. Biochem..

[6] Mei X., Liu X.Y., Zhou Y., Wang X.Q., Zeng L.T., Fu X.M., Li J.L., Tang J.C., Dong F., Yang Z.Y. (2017). Formation and emission of linalool in tea (*Camellia sinensis*) leaves infested by tea green leafhopper (*Empoasca* (*Matsumurasca*) *onukii* Matsuda. Food Chem..

[7] Fu X.M., Chen Y.Y., Mei X., Katsuno T., Kobayashi E., Dong F., Watanabe N., Yang Z.Y. (2015). Regulation of formation of aroma compounds of tea (*Camellia sinensis*) leaves by single light wavelength. Sci. Rep..

[8] Yang Z.Y., Kobayashi E., Katsuno T., Asanuma T., Fujimori T., Ishikawa T., Tomomura M., Mochizuki K., Watase T., Nakamura Y. (2012). Characterisation of volatile and non-volatile metabolites in etiolated leaves of tea (*Camellia sinensis*) plants in the dark. Food Chem..

[9] Gui J.D., Fu X.M., Zhou Y., Katsuno T., Mei X., Deng R.F., Xu X.L., Zhang L.Y., Dong F., Watanabe N. (2015). Does enzymatic hydrolysis of glycosidically bound aroma compounds really contribute to the formation of aroma compounds during the oolong tea manufacturing process?. J. Agric. Food Chem..

[10] Zeng L.T., Zhou Y., Gui J.D., Fu X.M., Mei X., Zhen Y.P., Ye T.X., Du B., Dong F., Watanabe N. (2016). Formation of volatile tea constituent indole during the oolong tea manufacturing process. J. Agric. Food Chem..

[11] Zhou Y., Zeng L.T., Liu X.Y., Gui J.D., Mei X., Fu X.M., Dong F., Tang J.C., Zhang L.Y., Yang Z.Y. (2017). Formation of (*E*)-nerolidol in tea (*Camellia sinensis*) leaves exposed to multiple stresses during tea manufacturing. Food Chem..

[12] Zeng L.T., Zhou Y., Fu X.M., Mei X., Cheng S.H., Gui J.D., Dong F., Tang J.C., Ma S.Z., Yang Z.Y. (2017). Does oolong tea (*Camellia sinensis*) made from a combination of leaf and stem smell more aromatic than leaf-only tea? Contribution of the stem to oolong tea aroma. Food Chem..

[13] Zeng L.T., Zhou Y., Fu X.M., Liao Y.Y., Yuan Y.F., Jia Y.X., Dong F., Yang Z.Y. (2018). Biosynthesis of jasmine lactone in tea (*Camellia sinensis*) leaves and its formation in response to multiple stresses. J. Agric. Food Chem..

[14] Dudareva N., Klempien A., Muhlemann J.K., Kaplan I. (2013). Biosynthesis, function and metabolic engineering of plant volatile organic compounds. New Phytol..

[15] Dong F., Yang Z.Y., Baldermann S., Sato Y., Asai T., Watanabe N. (2011). Herbivore-induced volatiles from tea (*Camellia sinensis*) plants and their involvement in intraplant communication and changes in endogenous nonvolatile metabolites. J. Agric. Food Chem..

[16] Zeng L.T., Liao Y.Y., Li J.L., Zhou Y., Tang J.C., Dong F., Yang Z.Y. (2017). α-Farnesene and ocimene induce metabolite changes by volatile signaling in neighboring tea (*Camellia sinensis*) plants. Plant Sci..

[17] Wasternack C., Hause B. (2013). Jasmonates: Biosynthesis, perception, signal transduction and action in plant stress response, growth and development. An update to the 2007 review in Annals of Botany. Ann. Bot..

[18] Song W.C., Brash A.R. (1991). Purification of an allene oxide synthase and identification of the enzyme as a cytochrome P-450. Science.

[19] Song W.C., Funk C.D., Brash A.R. (1993). Molecular cloning of an allene oxide synthase: A cytochrome P-450 specialized for metabolism of fatty acid hydroperoxides. Proc. Natl. Acad. Sci. USA.

[20] Turner J.G., Ellis C., Devoto A. (2002). The jasmonate signal pathway. Plant Cell.

[21] Peng Q.Y., Zhou Y., Liao Y.Y., Zeng L.T., Xu X.L., Jia Y.X., Dong F., Li J.L., Tang J.C., Yang Z.Y. (2018). Functional characterization of an allene oxide synthase involved in biosynthesis of jasmonic acid and its influence on metabolite profiles and ethylene formation in tea (*Camellia sinensis*) flowers. Int. J. Mol. Sci..

[22] Dong F., Zeng L.T., Yu Z.M., Li J.L., Tang J.C., Su X.G., Yang Z.Y. (2018). Differential accumulation of aroma compounds in normal green and albino-induced yellow tea (*Camellia sinensis*) leaves. Molecules.

[23] Wang K.R., Li M., Zhang L.J., Liang Y.R. (2015). Studies on classification of albino tea resources. J. Tea.

[24] Wang W., Guo Y.L. (2017). Development and application of albino tea varieties. J. Food Safety Quality.

[25] Wang K.K., Li N.N., Du Y.Y., Liang Y.R. (2013). Effect of sunlight shielding on leaf structure and amino acids concentration of light sensitive albino tea plan. Afr. J. Biotechnol..

[26] Song L., Ma Q., Zou Z., Sun K., Yao Y., Tao J., Kaleri N.A., Li X. (2017). Molecular Link between Leaf coloration and gene expression of flavonoid and carotenoid biosynthesis in *Camellia sinensis* cultivar ‘Huangjinya’. Front. Plant Sci..

[27] Liu G.F., Han Z.X., Feng L., Gao L.P., Gao M.J., Gruber M.Y., Zhang Z.L., Xia T., Wan X.C., Wei S. (2017). Metabolic flux redirection and transcriptomic reprogramming in the albino tea cultivar ‘Yu-Jin-Xiang’ with an emphasis on catechin production. Sci. Rep..

[28] Cheng S.H., Fu X.M., Liao Y.Y., Xu X.L., Zeng L.T., Tang J.C., Li J.L., Lai J.H., Yang Z.Y. (2019). Differential accumulation of specialized metabolite L-theanine in green and albino-induced yellow tea (*Camellia sinensis*) leaves. Food Chem..

[29] Pichersky E., Noel J.P., Dudareva N. (2006). Biosynthesis of plant volatiles: nature’s diversity and ingenuity. Science.

[30] Liu G.F., Liu J.J., He Z.R., Wang F.M., Yang H., Yan Y.F., Gao M.J., Gruber M.Y., Wan X.C., Wei S. (2018). Implementation of CsLIS/NES in linalool biosynthesis involves transcript splicing regulation in *Camellia sinensis*. Plant Cell Environ..

[31] Rhee S., Parris K.D., Ahmed S.A., Miles E.W., Davies D.R. (1996). Exchange of K^+^ or Cs^+^ for Na^+^ induces local and long-range changes in the three-dimensional structure of the tryptophan synthase α_2_β_2_ complex. Biochemistry.

[32] Zhu J.Y., Wang X.W., Guo L.X., Xu Q.S., Zhao S.Q., Li F.D., Yan X.M., Liu S.R., Wei C.L. (2018). Characterization and alternative splicing profiles of lipoxygenase gene family in tea plant (*Camellia sinensis*). Plant Cell Physiol..

[33] Deng W.W., Wu Y.L., Li Y.Y., Tan Z., Wei C.L. (2016). Molecular cloning and characterization of hydroperoxide lyase gene in the leaves of tea plant (*Camellia sinensis*). J. Agric. Food Chem..

[34] Negre-Zakharov F., Long M.C., Dudareva N., Osbourn A.E., Lanzotti V. (2009). Floral scents and fruit aromas inspired by nature. Plant-Derived Natural Products.

[35] Dombrecht B., Xue G.P., Sprague S.J., Kirkegaard J.A., Ross J.J., Reid J.B., Fitt G.P., Sewelam N., Schenk P.M., Manners J.M. (2007). MYC2 differentially modulates diverse jasmonate-dependent function in *Arabidopsis*. Plant Cell.

[36] Hao X., Horvath D.P., Chao W.S., Yang Y., Wang X., Xiao B. (2014). Identification and evaluation of reliable reference genes for quantitative real-time PCR analysis in tea plant (*Camellia sinensis* (L.) O. Kuntze). Int. J. Mol. Sci..

