# Supplementary material for: Influence of Chloroplast Defects on Formation of Jasmonic Acid and Characteristic Aroma Compounds in Tea (Camellia sinensis) Leaves Exposed to Postharvest Stresses
Source: Int J Mol Sci. 2019 Feb 27;20(5):1044. doi: 10.3390/ijms20051044 (PMC6429154; doi:10.3390/ijms20051044)
Supplement: Supplementary file 1 [file ijms-20-01044-s001.pdf]

SUPPLEMENTARY INFORMATION

Influence of Chloroplast Defects on Formation of Jasmonic Acid and Characteristic Aroma Compounds in Tea (*Camellia sinensis*) Leaves Exposed to Postharvest Stresses

Jianlong Li [1,†], Lanting Zeng [2,3,†], Yinyin Liao [2,3], Dachuan Gu [2,3], Jinchi Tang [1,*], Ziyin Yang [2,3,*]

[1] Tea Research Institute, Guangdong Academy of Agricultural Sciences & Guangdong Provincial Key Laboratory of Tea Plant Resources Innovation and Utilization, Dafeng Road 6, Tianhe District, Guangzhou 510640, China; skylong.41@163.com (J.L.)

[2] Guangdong Provincial Key Laboratory of Applied Botany & Key Laboratory of South China Agricultural Plant Molecular Analysis and Genetic Improvement, South China Botanical Garden, Chinese Academy of Sciences, Xingke Road 723, Tianhe District, Guangzhou 510650, China; zenglanting@scbg.ac.cn (L.Z.); honey_yyliao@scbg.ac.cn (Y.L.) ; gdcawang@126.com (D.G.)

[3] College of Advanced Agricultural Sciences, University of Chinese Academy of Sciences, No. 19A Yuquan Road, Beijing 100049, China

† These authors contributed equally to this work.

* Correspondence: tangjinchi@126.com; Tel.: +86-20-8516-1049 (J.T.) ; zyyang@scbg.ac.cn; Tel.: +86-20-3807-2989 (Z.Y.)

Table S1. The primers used for quantitative real time PCR (qRT-PCR) in the study.

	Accession number	Forward primer 5'-3'	Reverse primer 5'-3'
CsEF1	KA280301.1	TTGGACAAGCTCAAGGCTGAACG	ATGGCCAGGAGCATCAATGACAGT
CsLOX1	EU195885	GCTGACTGGACAACCGATGA	CAACATATGCTTCTATGAAAATGC
CsLOX2	FJ418174	GTTTTGTCAAATCATTCGGT	TTCTTCAAACTCAAGTTTG
CsLOX3	FJ794853	GGGACAACACTGTATGGG	CCAGAGTCATGAGCAAGGG
CsLOX4	MG708225	ACTTGAAGAATAGATGTGGAGC	TCTTACAAATGCATTCACTTCTC
CsLOX6	MG708227	GACCCAAGCCTCACAAATAG	GCTTCATTTATGCTACTCACAC
CsLOX7	MG708228	ATTTCTCTTCTCTCACTCTCAC	GAACACCTCTCCATCACACT
CsAOS2	AHY03308.1	GTTTCAACCGTTTGCGACCA	GTTTCAACCGTTTGCGACCA
CsAOC	GEFQ01049691.1	GCAGAAGCGGTTGGGAAT	CACCAGTCACAGCGAGATAC
CsOPR1	KA281144.1	ACTTGTCTGTAGAGGAGATTGGA	AACCGATTGTAGATGCTGTTCAT
CsOPR2	GEFQ01093164.1	ATGTTCCACCACCTCCAGTAT	TCATCTACGAGCAAGCCTATATCA
CsNES1	KY033151	CAGCACAAACGAAATTTCCT	CATTCCATGACCCAAGAGAA
CsNES2	-	GAATGACAATCCAGGCATTG	TGGTGAGAATGGATTTGGAG
CsLIS	KF006849	TCCAACCCCTCAATACAGAAAGACTATC	TTGGCTTTGTAGAAGTGCTTCAATCTC
CsTSA	KX022968	ACCACACCTACTACTCCAACA	CTTACAGATACACGAGCACCAG
CsTSB2	KX022970	CCTTATCTCCACGCCCACTA	ACGACTATGCCGACTTGAAG
CsHPL	GW342656	ATCCCTAACACCGCCATCG	CCTTGGAACCAGAAGTAGTC
CsMYC2a	KU892079	ATCCCGGTTTTCAGGTCCAC	ATTCGAATCATCGCGTCCCA
CsMYC2b	KU892080	TTGCCCTTTGGATACCCACC	TTCGCGTGAAAATGCTGCAA
CsMYC2c	KU892081	TGCAACAAGCCAAGTCACTG	AGCTCAGATTCGGCATTGGT

EF1, encoding elongation factor 1; LOX, lipoxygenase; AOC, allene oxide cyclase; AOS, allene oxide synthase; OPR, 12-oxo-phytodienoic acid reductase; NES, (E)-nerolidol synthase; LIS, linalool synthase; TSA, tryptophan synthase α-subunit; TSB, tryptophan synthase β-subunit; HPL, hydroperoxide lyase.

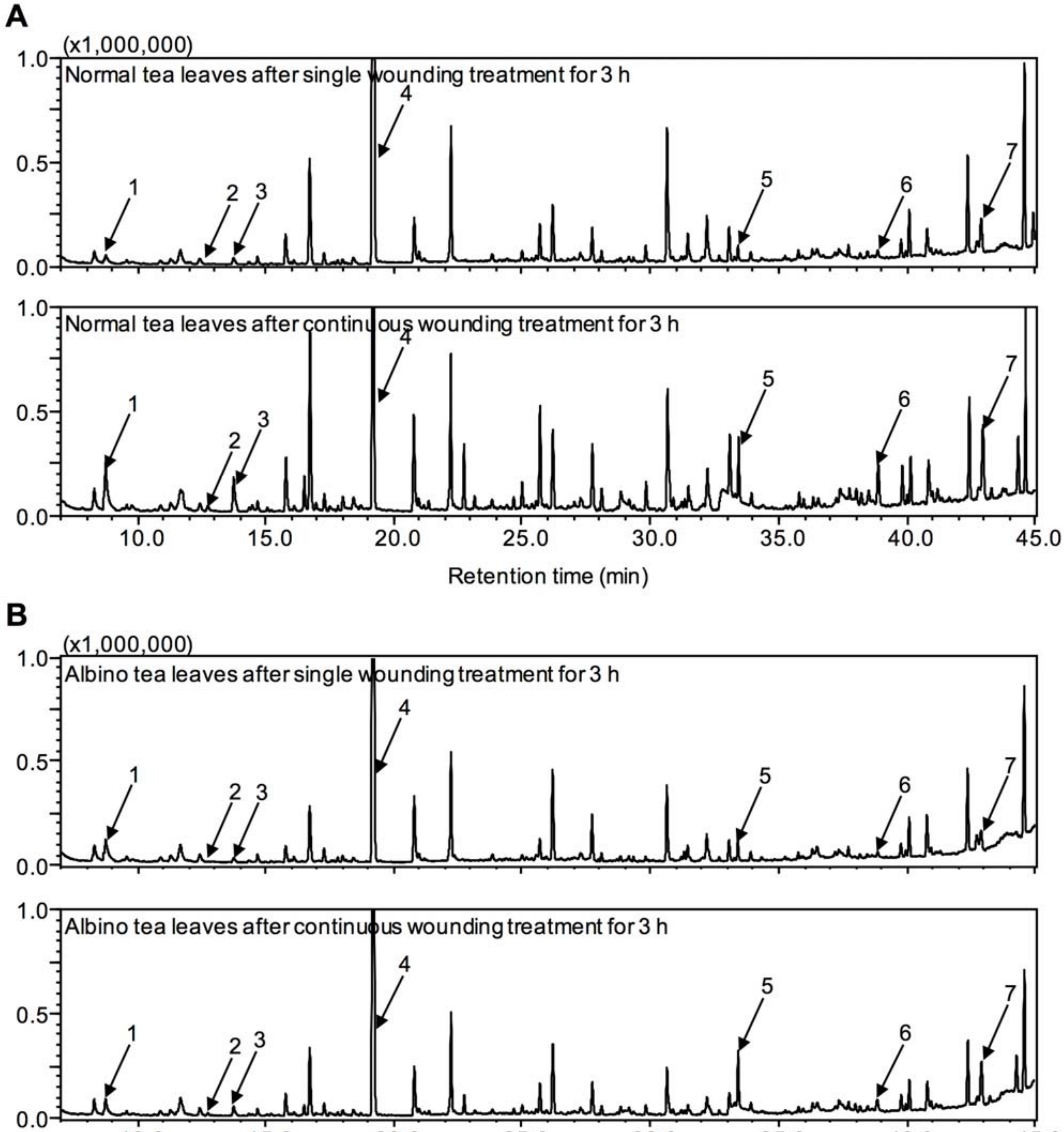

Figure S1. GC-MS chromatograms of aroma compounds in normal tea leaves (A) and albino tea leaves (B) exposed to single wounding treatment and continuous wounding treatment. 1, 2-Hexenal; 2, 1-Hexenol; 3, (*Z*)-3-Hexenol; 4, Linalool; 5, (*E*)-Nerolidol; 6, Jasmine lactone; 7, Indole. These aroma compounds were investigated in the study.
